# Eriocitrin inhibits epithelial-mesenchymal transformation (EMT) in lung adenocarcinoma cells via triggering ferroptosis

**DOI:** 10.18632/aging.205049

**Published:** 2023-10-02

**Authors:** Minglang Gao, Kai Lai, Yu Deng, Zilong Lu, Congkuan Song, Wenjie Wang, Chenzhen Xu, Ning Li, Qing Geng

**Affiliations:** 1 Department of Thoracic Surgery, Renmin Hospital of Wuhan University, Wuhan 430060, China; 2 Department of Cardiothoracic Surgery, The Second Affiliated Hospital of Nanchang University, Nanchang 330000, China

**Keywords:** eriocitrin, LUAD, EMT, ferroptosis, metastasis, chemopreventive

## Abstract

Introduction: Lung adenocarcinoma (LUAD) is the most prevalent pathological subtype of non-small cell lung cancer (NSCLC), characterized by a high propensity for relapse and metastasis due to epithelial-mesenchymal transition (EMT) of cancer cells. Ferroptosis, a newly discovered regulated cell death modality, is interconnected with the EMT process in certain cancers. Eriocitrin, a natural flavonoid compound, exerts anti-inflammatory and anticancer effects.

Objectives: The aim of this study is to investigate the potential inhibitory effect of eriocitrin on lung adenocarcinoma metastasis and explore whether its underlying mechanism involves ferroptosis induction in cancer cells.

Methods: The CCK8 assay and wound healing assay and transwell were conducted to determine the cell viability and migration ability of A549 and H1299 cells, respectively. EMT process was assessed by western blot and RT-PCR to detect protein and mRNA levels of EMT markers. ROS and cell iron were measured to determine ferroptosis level.

Results: Eriocitrin treatment significantly inhibited cell viability and migration ability in a concentration-dependent manner. Furthermore, eriocitrin administration for 24 hours resulted in enhanced expression of E-cadherin, while downregulating vimentin, N-cadherin and snail expression, indicating marked repression of the EMT process. Additionally, eriocitrin significantly induced ferroptosis in A549 and H1299 cells, as evidenced by increased ROS levels, downregulation of Nrf-2, SLC7A11 and GPX4 expression, and enhanced cellular iron accumulation. Moreover, pretreatment with the ferroptosis inhibitor ferrostatin-1 effectively abrogated the inhibitory effects of eriocitrin on EMT.

Conclusions: Our findings further support the anti-cancer properties of eriocitrin, as evidenced by its ability to inhibit the EMT process in LUAD cells, which is partially mediated through induction of ferroptosis in cancer cells.

## INTRODUCTION

Lung cancer is still the most prevailing cancer type in the world with high rates of cancer-related deaths, and its major subtype is non-small cell lung cancer (NSCLC), accounting for approximately 85% diagnosed cases [1, 2]. Lung adenocarcinoma (LUAD) is the most common pathological pattern of NSCLC, which converts readily to metastasis and recrudesce. Currently, in the clinical practice, the management strategies for LUAD involve curative surgical resection of tumor, combined with chemotherapy, targeted-therapy and immunotherapy. However, resistance and side effects of chemotherapy, as well as limited indications for targeted-therapy and immunotherapy severely restrict their efficacy [3, 4]. Therefore, there is an urgent need to explore safer alternatives like natural compounds for intervening the progression of LUAD.

Epithelial-mesenchymal transition (EMT) is a continuous process which is critical for embryonic morphogenesis by affecting cell migration [5]. During EMT process, cells cast off their epithelial nature and gain mesenchymal characteristics step by step, as manifested by destruction of the integrity of the intercellular junction, which is readily turned motile [6]. Given this nature of EMT, it is not hard to link cancer progression with this process. Growing body of evidence have indicated that cancer cells can hijack EMT to facilitate tumorigenesis, invasion and metastasis, maintain tumor cells stemness, resist to tumor therapy and confer tumor plasticity [6, 7]. Undoubtedly, lung epithelial cells undergo EMT to adopt mesenchymal properties, participating in carcinogenesis and subsequent cancer development. Targeting EMT process is reported to blunt lung cancer cells migration [8]. What’s more, LUAD cells rely on EMT to become plastic to resist EGFR-TKIs [9]. Therefore, inhibiting EMT process might be a potential and effective prevention for intervening LUAD progression.

Ferroptosis is a newly discovered regulatory nonapoptotic cell death form. The core mechanism of ferroptosis is closely related to iron-dependent lipid peroxidation, which causes plasma membrane destruction and releases cellular contents [10]. Some studies have revealed that stimulating ferroptosis is to some extent likely suppressing EMT in several cancers [11–13]. Intriguingly, however, the relationship between ferroptosis and EMT in LUAD deserves to be explored.

**Figure 1:**
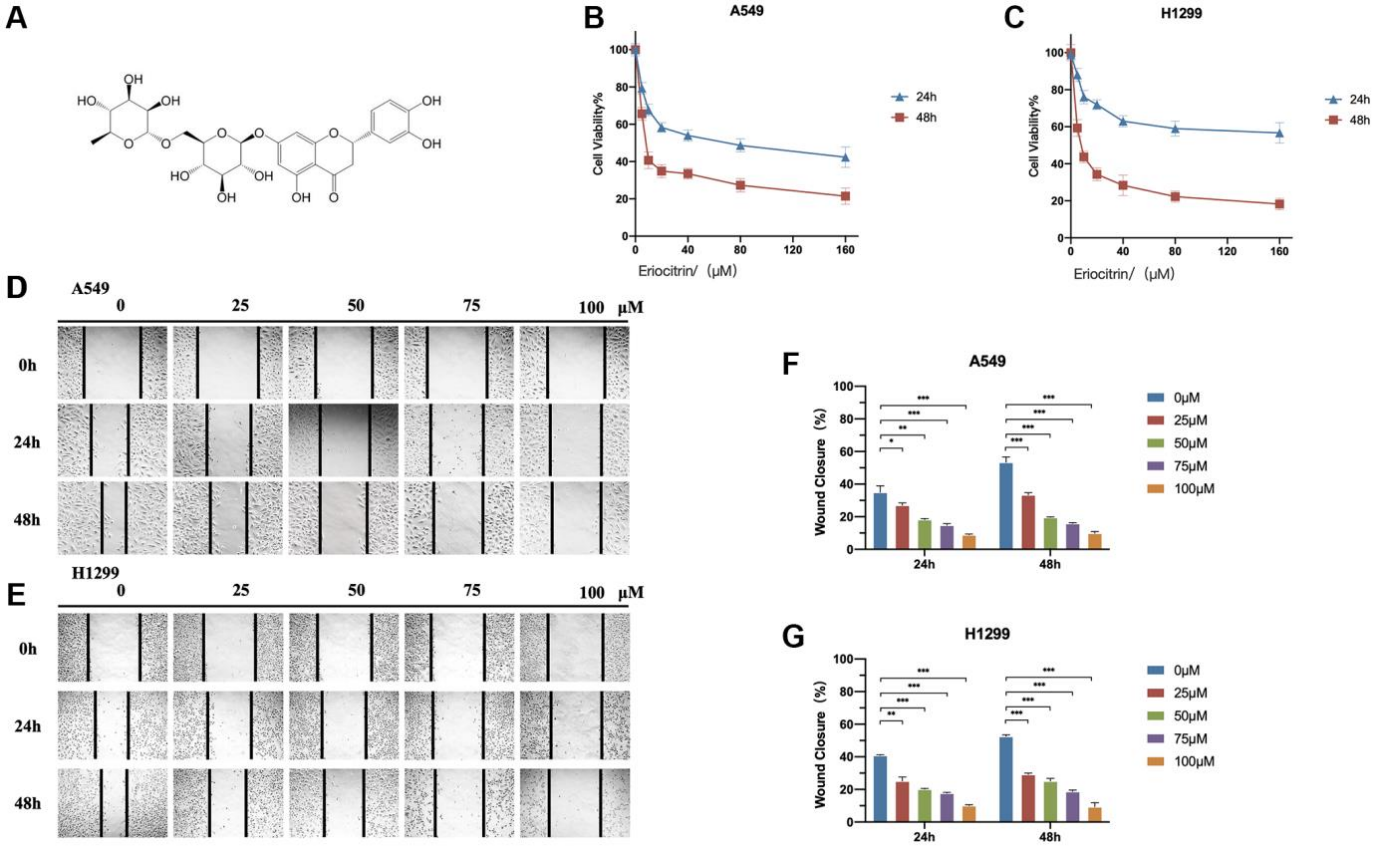
**Eriocitrin treatment attenuated the viability and invasion of A549 and H1299 cells in a concentration-dependent manner.** (**A**) The molecular structure of eriocitrin. (**B**, **C**) CCK8 assay. Cell viability of A549 and H1299 cells were measured after treatment of eriocitrin at various concentrations (0, 5, 10, 20, 40, 80, and 160 μM) for 24 h or 48 h. (**D**, **E**) Wound healing assay showed the migration ability of A549 and H1299 cells treated with eriocitrin at different concentrations (0, 25, 50, 75 and 100 μM) for 24 h or 48 h; (**F**, **G**) Wound closure ratio of A549 and H1299 cells after treatment with eriocitrin. ^*^P < 0.05, ^**^P < 0.01, and ^***^P < 0.001.

Eriocitrin (Figure 1A), derived from lemon and citrate juice, is a natural flavonoid compound. It has been reported to exert multiple biological functions in distinct settings *in vitro*, such as anti-inflammatory [14, 15], anti-diabetic [16] and lowering-lipid effects [17, 18]. Besides, emerging lines of evidence suggest that eriocitrin could inhibit cancer cells proliferation via diverse mechanics. Wang Z et al. founded that eriocitrin could stimulate liver carcinoma cells apoptosis by activating mitochondrial-mediated apoptotic singling pathway [19]. Apart from that, recent study showed that eriocitrin strongly promotes ROS generation and thus to induce breast cancer cells apoptosis via STAT3 pathway [20]. However, little is known about its role in LUAD.

Herein, we discovered that eriocitrin restrained cell viability and EMT process in LUAD cells in a concentration-dependent manner. Besides, eriocitrin triggered ferroptosis via enhancing cellular ROS level, which is mediated by the downregulation of Nrf-2. Inhibition of ferroptosis by administrating Ferrostatin-1 abrogated the beneficial effects of eriocitrin. Taken together, we suggest that eriocitrin might be a promising chemo-preventive remedy for LUAD tumorigenesis and metastasis.

## MATERIALS AND METHODS

### Reagents and antibodies

Eriocitrin was purchased from MCE (Shanghai, China). Eriocitrin stock solution was prepared in 0.05% DMSO and cells were treated with different concentrations of Eriocitrin using micropipettes. Primary antibodies against Nrf2, GPX4, SLC7A11, FTH1, N-cadherin, E-cadherin, Vimentin were purchased from Proteintech Group (Wuhan, China). Iron assay kit were purchased from Jiancheng Bioengineering Institute (Nanjing, China). The ROS Fluorescent Probe Kit (2′7-dichlorofluorescin-diacetate, DCFH-DA) was used to detect ROS of cell (Biosharp, China). The CCK8 assay kit were purchased from Biosharp, China. The primers for Nrf2, GPX4, SLC7A11, FTH1, N-cadherin, E-cadherin, Snail, GAPDH were purchased from Servicebio, China.

### Cell culture

The human-type II cell alveolar epithelial cell line A549 and human lung adenocarcinoma cell line H1299 were obtained from the American Type Culture Collection (ATCC, Manassas VA, USA). A549 and H1299 were cultured in Ham’s F12K culture medium (Servicebio, China) and RPMI 1640 culture medium (Servicebio, China) respectively, both supplemented with 10% fetal bovine serum (FBS; Gibco, USA) and 1% penicillin/streptomycin (P/S) (Biosharp, China), and maintained at 37°C, 5% CO2 humidified incubator.

### CCK8 assay

Cells were seeded into 96-well plates with 100 μl culture medium. After 24 h of cultivation, different concentrations of eriocitrin were added into plates for 0, 24 and 48 h. At indicated time, the cells were rinsed twice with PBS and 10 μl of CCK-8 solution was added to each well. After incubated for an hour at 37°C, the absorbances were examined at 450 nm according to CCK8 kit instruction.

### Migration assay

Migration was determined by wound healing assay. A549 and H1299 cells were cultured as monolayer in six-well plates. The bottom of each well was scratched using sterile 200 μl micropipette tip. Then, culture medium was removed and washed by 1× PBS twice. The monolayer cells were incubated with eriocitrin at non-toxic concentrations (0, 25, 50, 75, 100 M) at 37°C for 0, 24 and 48 h respectively. At indicated time points, migration was observed under a phase contrast microscope (Olympus) and images were captured. The wound area was determined by ImageJ software, and the percentage of wound closure was calculated.

### Cell invasion assay

Cell invasion abilities were assessed using a Transwell system, with A549 and H1299 cells seeded in the upper chamber. The lower chamber contained 500 μl of DMEM supplemented with 20% FBS and 50 μM eriocitrin. Following incubation for 24 hours, cells in the lower chamber were fixed with methanol and stained with crystal violet before counting the number of cells in three randomly selected fields viewed at 100× magnification.

### Detection of cell iron

The cell iron concentrations were determined according to manufacturer’s instruction. In short, petri dishes with cell density over 90% confluence was treated with DMSO or eriocitrin. After 24 h incubation, cells were lysed with RIPA and BCA method was used to detect total protein concentration. The blank tube, standard tube and under measured tubes were configured according to instruction and the absorbances were detected at 520 nm.

### Real-time fluorescence quantitative PCR

The total RNA of A549 and H1299 cells were extracted using TRIpure Total RNA Extraction Reagent (Biosharp, China) and cDNA was synthesized using EntiLink™ 1st Strand cDNA Synthesis Kit (Servicebio, China). Quantitative real-time PCR was performed using EnTurbo™ SYBR Green PCR SuperMix (Servicebio, China). The expression levels of target genes were uniformly normalized to GAPDH. All primers used in this study were listed in Supplementary Table 1.

### Western blotting

Cells were lysed in RIPA Lysis Buffer (Servicebio, China) contained with 1% Phenylmethanesulfonyl fluoride (PMSF, Servicebio, China). Proteins were separated on 10–15% SDS-polyacrylamide gradient gels and transferred onto PVDF membranes. The rapid block buff (Servicebio, China) was used to block non-specific binding for 30 minutes, and membranes were probed with primary antibodies in 4°C for 8–12 h, followed by incubation with anti-rabbit-HRP (1:5000; Proteintech, China) in 37°C for 1 h. GAPDH was selected as the internal reference. The protein bands were visualized with the enhanced chemiluminescence western blotting detection system (Bio-Rad, USA).

### ROS measurement

DHE was dissolved in DMSO to a final concentration of 5 mM and further diluted in phosphate-buffered saline (PBS, 1:1000) to a final DMSO concentration of 0.1%, which does not affect ROS generation. A549 and H1299 cells, seeded in a 6-well plate, were incubated by the DCFH-DA working solution (10 μM) at 37°C for 30 min. Finally, the fluorescence microscope was used to evaluate the level of ROS.

### Statistical analysis

All analyses were performed in SPSS 23.0 and GraphPad Prism 8 software. Data were demonstrated as mean ± standard deviation. Unless otherwise specified, the data were representative of at least three independent experiments, the two groups comparison was performed by student *t*-test and multiple group comparisons were performed by one-way analysis of variance (ANOVA). A confidence interval of 95% was used for all statistical tests, and *P* < 0.05 was regarded to be statistically significant.

### Availability of data and materials

The datasets used and/or analyzed during the current study are available from the corresponding author on reasonable request.

## RESULTS

### Eriocitrin treatment attenuated the proliferation and migration of A549 and H1299 cells in a concentration-dependent manner

**Figure 2:**
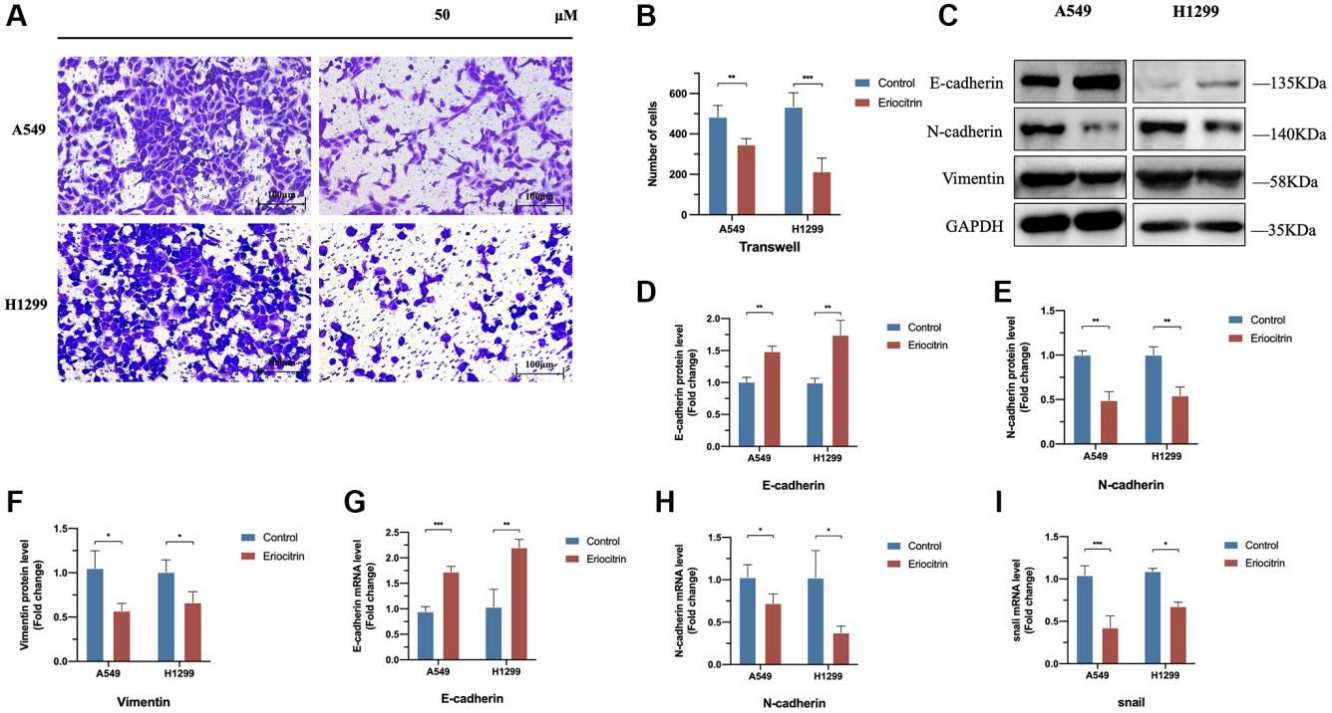
**Eriocitrin treatment strongly inhibited the EMT process of A549 and H1299 cells via abrogating Snail expression and inhibited the invasion.** (**A**, **B**) The transwell assay of A549 and h1299 cells and quantitative count after eriocitrin treatment 24 h at 50 μM concentration. (**C**–**F**) The protein expression of EMT-related genes after eriocitrin treatment 24 h in A549 and H1299 cells. (**G**–**I**) The mRNA expression of E-cadherin, N-cadherin, Snail in A549 and H1299 cells after eriocitrin treatment 24 h at 50 μM concentration. ^*^P < 0.05, ^**^P < 0.01, and ^***^P < 0.001.

In order to determine whether eriocitrin exhibits anti-tumor effects in LUAD, we treated A549 and H1299 cells with eriocitrin in various concentrations and detected the cell viability and wound healing ratio at indicated timepoints. As is shown in Figure 1B, 1C, the cell viability was markedly reduced by eriocitrin both in A549 and H1299 cells, and presenting in a concentration-dependent manner. Furthermore, the migratory ability of A549 and H1299 was also repressed, as indicated by the decreased wound closure caused by eriocitrin administration (Figure 1D–1G). Consistent with cell viability, the higher concentration eriocitrin was, the more obvious inhibition was exerted in cell migration. Next, we assessed the invasive potential of A549 and H1299 cells treated with eriocitrin using transwell assays. Compared to the untreated groups, eriocitrin exhibited significantly reduced cell invasion rates (Figure 2A, 2B). These results strongly supported that eriocitrin suppresses proliferation, invasion, and migration of A549 and H1299 cells *in vitro*.

### Eriocitrin treatment suppressed the EMT process both in A549 and H1299 cells

Previous studies have reported EMT plays a critical role in cancer invasion and migration [6]. We hypothesize that the inhibitory effects to cancer cells invasion and migration exerted by eriocitrin are at least partially mediated by suppressing EMT process. Therefore, we treated A549 and H1299 cells with 50 μM eriocitrin 24 hours and detected the EMT markers expression. Our results showed that E-cadherin was upregulated, while N-cadherin and vimentin were downregulated after eriocitrin exposure (Figure 2C–2F). In addition, the mRNA of E-cadherin and N-cadherin exhibited the same changes as their protein variation triggered by eriocitrin (Figure 2G, 2H). Furthermore, we found that the mRNA of snail, a well-known transcriptional inhibitor of E-cadherin, was markedly abrogated in A549 cells and slightly inhibited in H1299 cells after treated with eriocitrin compared to the control (Figure 2I). These results demonstrated that eriocitrin blunt the EMT process of lung cancer cells, which may be partially explained to depress lung cancer cells invasion and migration.

### Eriocitrin treatment triggered ferroptosis in A549 and H1299 cells by suppressing Nrf2 expression

**Figure 3:**
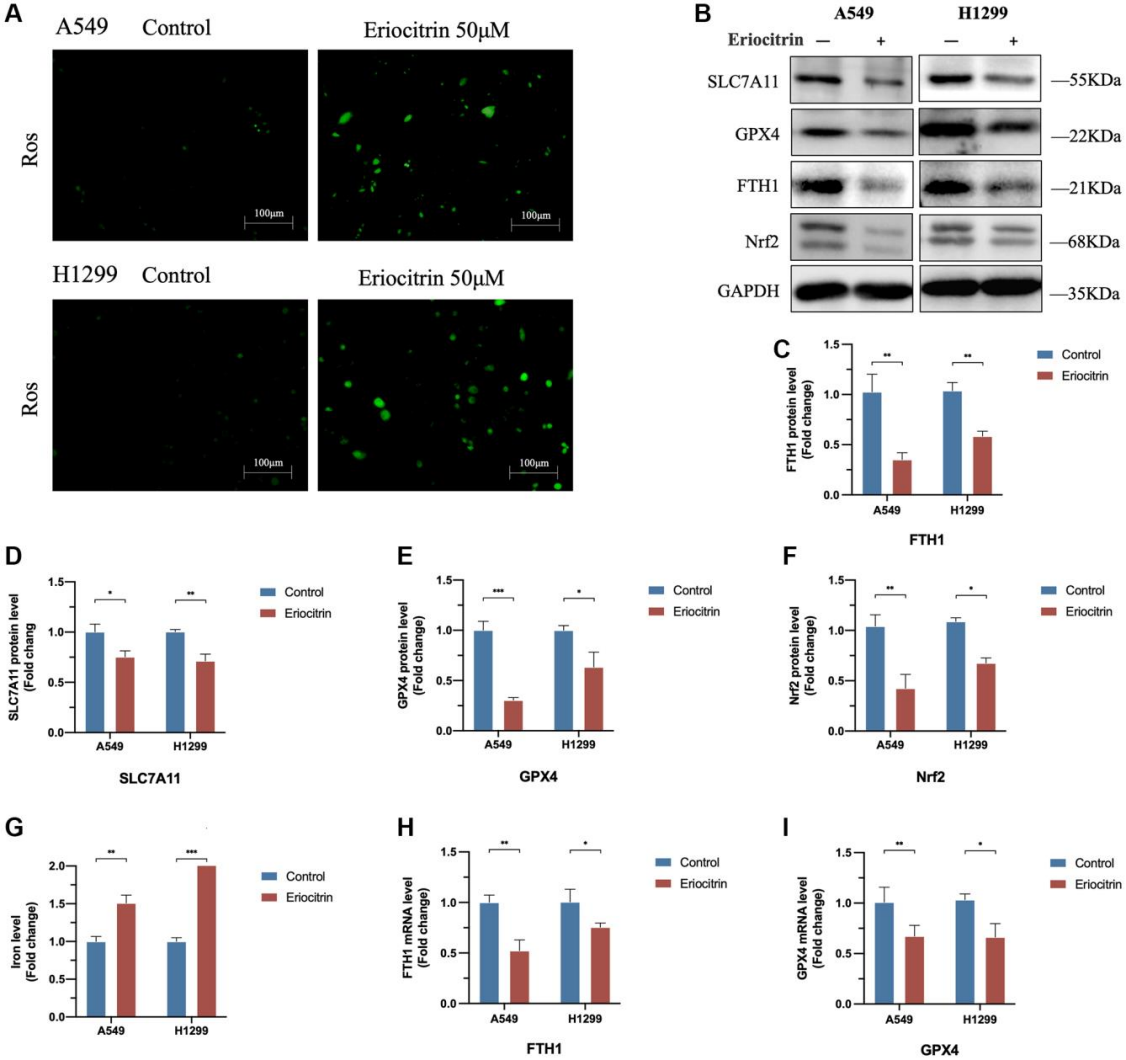
**Eriocitrin treatment triggered ferroptosis in A549 and H1299 cells via suppressing Nrf2 expression.** (**A**) Fluorescent images of ROS in A549 and H1299 cells. (**B**–**F**) Protein expression of ferroptosis-related genes after 50 μM concentration eriocitrin treatment, including SLC7A11, GPX4, Nrf-2 and FTH1. (**G**) Relative iron levels of A549 and H1299 cells after 50 μM concentration eriocitrin treatment. (**H**, **I**) mRNA expression of ferroptosis-related genes after 50 μM concentration eriocitrin treatment, including GPX4 and FTH1. ^*^P < 0.05, ^**^P < 0.01, and ^***^P < 0.001.

As demonstrated above, the cell viability was significantly reduced via eriocitrin treatment, which prompted the proliferative ability was suppressed by eriocitrin or cell death was induced. A recent study indicates that eriocitrin can strongly stimulate ROS generation and cellular oxidative stress in breast cancer cells [20]. Given the peculiarity of oxidative death of ferroptosis, we assume that eriocitrin might provoke ferroptosis of lung cancer cells to suppress cell viability. As is shown in Figure 3A, in line with the previous investigation, the ROS was significantly enhanced by administrating eriocitrin in A549 and H1299 cells. What’s more, we detected the protein expression of ferroptosis indicator, encompassing SLC7A11, GPX4 and FTH1. As supposed, compared to control, we discovered that eriocitrin significantly downregulated SLC7A11, GPX4 and FTH1 expression both in A549 and H1299 cells (Figure 3B–3F). In addition, the mRNA of GPX4 and FTH1 exhibited the same changes as their protein variation triggered by eriocitrin (Figure 3H, 3I). Since ferroptosis is iron-dependent, we subsequently determined the cellular iron level. The results revealed that iron levels of A549 and H1299 cells were increased by eriocitrin treatment (Figure 3G). To certify the role of eriocitrin in the induction of ferroptosis, we blotted the Nrf-2 protein, an important modulator element for refraining ferroptosis [21], and uncovered the truth that eriocitrin downregulated Nrf-2 expression (Figure 3B, 3F). Above results illustrated that eriocitrin treatment stimulate lung cells ferroptosis, which might be mediated by lowering Nrf-2 expression.

### Ferroptosis inhibitor Ferrostatin1 abrogated the effects of eriocitrin on EMT processes of A549 and H1299 cells by impeding ferroptosis

**Figure 4:**
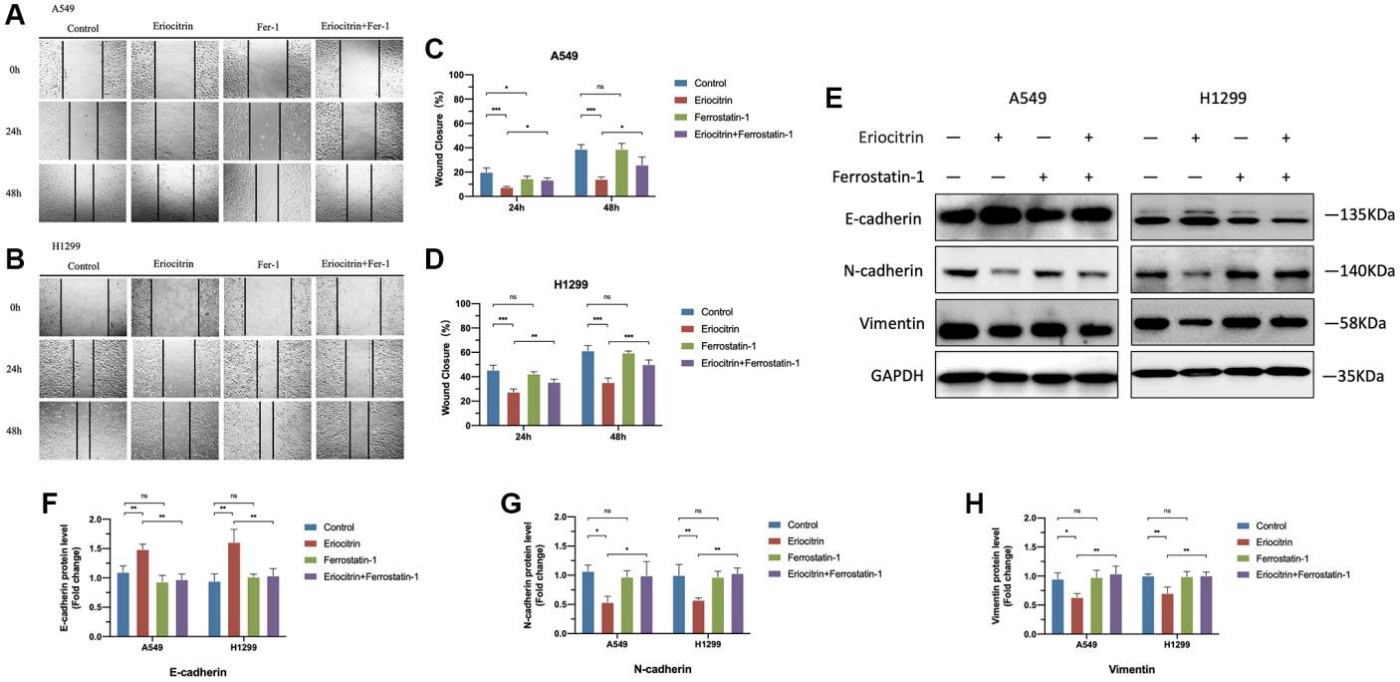
**Ferroptosis inhibitor Ferrostatin1 offseted the effects of eriocitrin on EMT processes of A549 and H1299 cells.** (**A**, **B**) Wound healing assay showed the migration ability of A549 and H1299 cells treated with 50 μM concentration eriocitrin, Fer-1, 50 μM concentration eriocitrin and Fer-l after 24 h. (**C**, **D**) Rate of wound closure of A549 and H1299 cells after treatment with eriocitrin, Fer-1, eriocitrin and Fer-1. (**E**–**H**) Western blotting assay of EMT related protein expression after Eriocitrin treatment. ^*^P < 0.05, ^**^P < 0.01, and ^***^P < 0.001. Abbreviation: Fer-1: Ferrostatin1.

**Figure 5:**
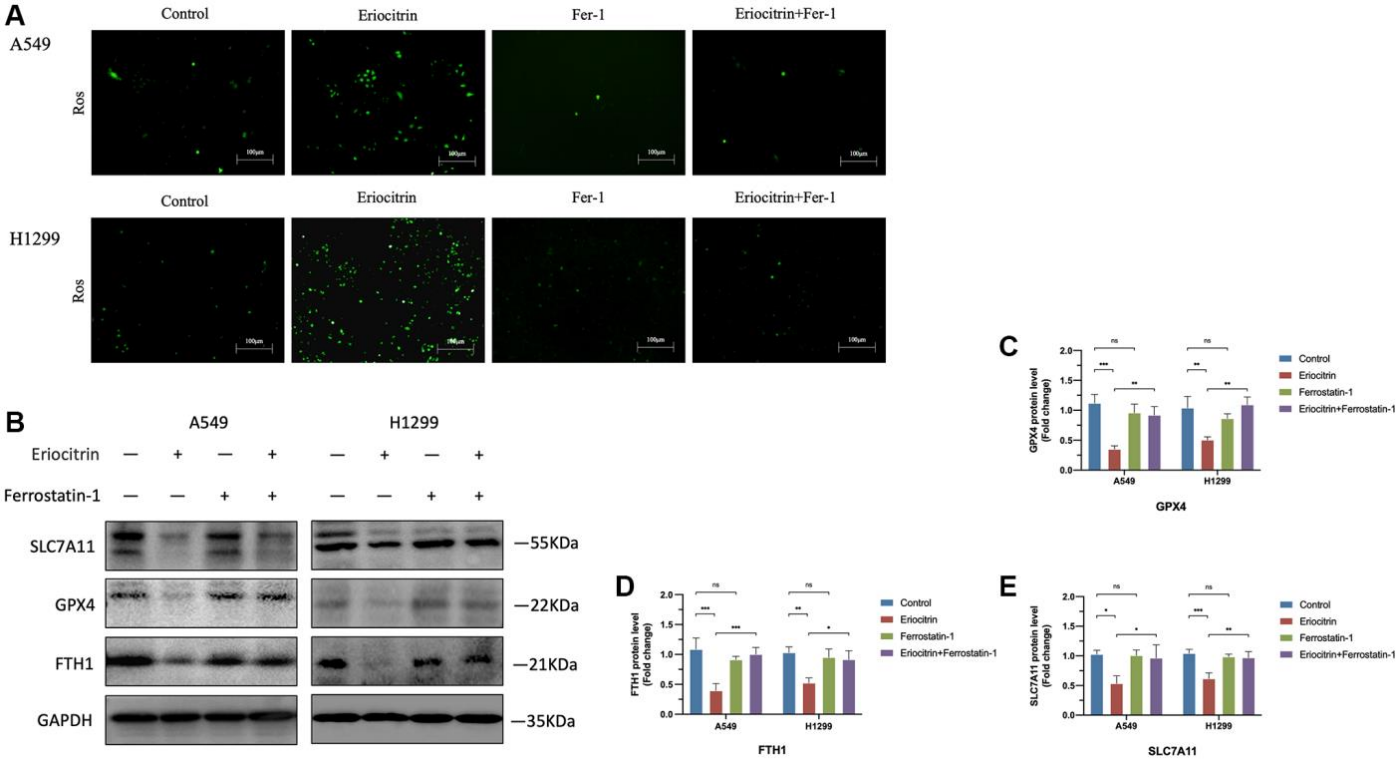
**Ferroptosis inhibitor Ferrostatin1 impeded the effects of eriocitrin on ferroptosis A549 and H1299 cells.** (**A**) Fluorescent images of ROS in A549 and H1299 cells after treatment with 50 μM concentration eriocitrin, Fer-1, 50 μM concentration eriocitrin and Fer-1. (**B**–**E**) Western blotting assay of ferroptosis related protein expression after Eriocitrin treatment. ^*^P < 0.05, ^**^P < 0.01, and ^***^P < 0.001. Abbreviation: Fer-1: Ferrostatin1.

To further determine the role of ferroptosis played in EMT process, we simultaneously treated cells with ferroptosis inhibitor Ferrostatin1 (Fer1) after administrating 50 μM concentration eriocitrin. The wound healing assay demonstrated that Fer1 diminished the inhibitory effects of eriocitrin to cancer cell migration, as manifested by enhanced wound closure ratio compared to treating 50 μM concentration eriocitrin alone in a time-dependent manner (Figure 4A–4D). Fer1 treatment alone exerted no significant effects to cancer cell migration by contrast to control (Figure 4A–4D). It was worth to note that eriocitrin lessened the density of cultured cells by irritating cell ferroptosis, which was also counteracted by Fer1. Next, we detected the protein expression of EMT markers after Fer1 treatment. As is shown in Figure 4E–4H, increased E-cadherin expression, decreased N-cadherin and vimentin expression were observed in the eriocitrin combined Fer1 group compared to treating eriocitrin alone both in A549 and H1299 cells, which implicated that the EMT process was partially hindered. Besides, Fer1 intensively increased cellular ROS level, reversed the expression of biomarkers of ferroptosis restrained by eriocitrin treatment (Figure 5A–5E). These results suggested that eriocitrin blunted EMT process of lung cancer cells through inducing ferroptosis and inhibiting ferroptosis could dwindle the effects.

## DISCUSSION

Lung cancer remains the leading cause of cancer-related deaths globally, in which LUAD is the utmost among them. Despite the soaring advent of cancer remedy consisting of curative tumor resection, immunotherapy, targeted-therapy, chemotherapy and radiotherapy, it remains largely unsatisfactory with patient prognosis who is suffering LUAD due to recurrence and metastasis. Therefore, it deserves to explore novel and effective chemopreventive agents. Eriocitrin, a natural flavonoid, has been demonstrated to exhibited various beneficial effects *in vitro* and *in vivo*. In this study, we discovered that eriocitrin could strongly inhibit lung cancer cells proliferation and effectively prevent lung cancer cells invasion and metastasis, which were abated by ferroptosis inhibitor Fer1.

EMT is the critical biological procedure during cancer metastasis and therapy resistance [22]. It should be noted that tumor metastasis is accounting for 90% deaths in patients with cancers [23]. Thus, swaying EMT process might be an optimal method with huge potential to prevent cancer metastasis and prolong patient survival. In our study, eriocitrin treatment obviously decreased the viability, deterred wound closure, upregulated epithelial biomarker (E-cadherin) expression, downregulated mesenchymal biomarkers (N-cadherin and vimentin) expression in A549 and H1299 cells, which are combined to convey the truth that eriocitrin hold promise to anti-cancer metastasis for anti-EMT procedure. Furthermore, snail is a well-known transcriptional regulator for inhibiting E-cadherin expression and key role for facilitating cancer EMT process [24], which was diminished under eriocitrin challenging. This strengthens the role of eriocitrin for anti-cancer metastasis. Our findings for the first time unravel the effect of eriocitrin to reverse EMT procedure in LUAD.

Eriocitrin displayed cytotoxicity to A549 and H1299 cells, which was determined by CCK8 assay. The cell viability was significantly depressed by eriocitrin in a concentration-dependent manner. This result is congruent with previous findings that dietary flavonoids possess strong anti-carcinogenic and anti-proliferative properties and reduce cancer risk [25]. In addition, eriocitrin has been evidenced to exhibit pharmacological action to suppress breast cancer cells and hepatocellular carcinoma cells proliferation through triggering apoptosis via diverse mechanisms [20, 26].

ROS is a cellular substance with two faces in biological and pathological conditions, which means that low level of ROS initiates a myriad of biological processes through activating signaling pathways called redox biology and excess ROS contributes to cell death denoting oxidative stress [27]. It has been documented that ROS functions both tumor-promoting and tumor-suppressing roles, and deregulating redox equilibrium strategy like ROS-inducing methods can be harnessed to kill cancer cells [28]. Intriguingly, although regarded as a natural plant-derived antioxidant, eriocitrin induced breast cancer cells apoptosis via exceeding cellular ROS production to cause cellular oxidative stress [20]. In line with this, our study further confirmed that pronouncing ROS production was promoting in A549 and H1299 cells in a relatively high concentration of 50 μM eriocitrin by inhibiting Nrf2 expression, which is a master antioxidative transcriptional factor. This prompts us eriocitrin plays a prooxidative role in lung cancer cells, which is not identical for its antioxidative properties. We suppose that opposite effects with respect to cellular oxidative stress in distinct pathological settings may be attribute to different concentration of eriocitrin. As the truth is that exogenous antioxidants could display double-edge sword actions, namely high dose reacts to prooxidative cytotoxicity, while low dose exhibits antioxidative protective effects [29]. Previous studies focus on ROS-induced cell apoptosis [20, 26], in this study, we primary reported that eriocitrin triggered ferroptosis of lung cancer cells, which is a ROS-dependent cell death form. After incubated with eriocitrin, ferroptotic biomarkers SLC7A11, GPX4 and FTH1 were significantly downregulated both in A549 and H1299 cells. Furthermore, iron accumulation level of lung cancer cells was intensively increased by eriocitrin. All the findings indicated that eriocitrin could give rise to ferroptosis-inducing effects. Alternatively, it strengthens the notion that ferroptosis can be irritated to exert potent antitumor activity [30, 31].

Several lines of studies have indicated that cancer cells undergoing EMT are more sensitive and vulnerable to ferroptosis [13, 32, 33]. What’s more, Liu L reported that SIRT3, a mitochondrial NAD^+^-dependent histone deacetylase, led gallbladder cancer cells to ferroptosis, thus to function for EMT inhibition and tumor suppression [34]. Similarly, 6-Gingerol, a bioactive plant-derived compound, was evidenced to decreased EMT-related protein expression in prostate cancer cells mediated by ferroptosis-inducing cell death. Co-treatment with the ferroptosis inhibitor, ferrostatin-1, reversed the beneficial inhibitory effects of 6-Gingerol to EMT [11]. In accordance with the above studies, we discovered that EMT process was blunted following eriocitrin exposure and this effect was significantly rescued by Fer1. Otherwise, Fer1 also attenuated the ROS content and salvaged ferroptosis-related biomarkers expression, which reflected that the degree of ferroptosis was decreased. The underpinning mechanism needs further investigations.

Notably, there are several limitations in our study. Although we showed that snail expression was suppressed in transcriptional level, we did not detect other transcriptional factors that might mediating EMT, like twist. In addition, we supposed eriocitrin prevented EMT of lung cancer cells through luring ferroptosis, but the exact mechanism for ferroptosis linked to snail expression is not be illustrated and need to be addressed. Last but not least, we do not convey the beneficial effects of eriocitrin into vivo study and verify the findings obtained in our vitro study.

## CONCLUSIONS

**Figure 6:**
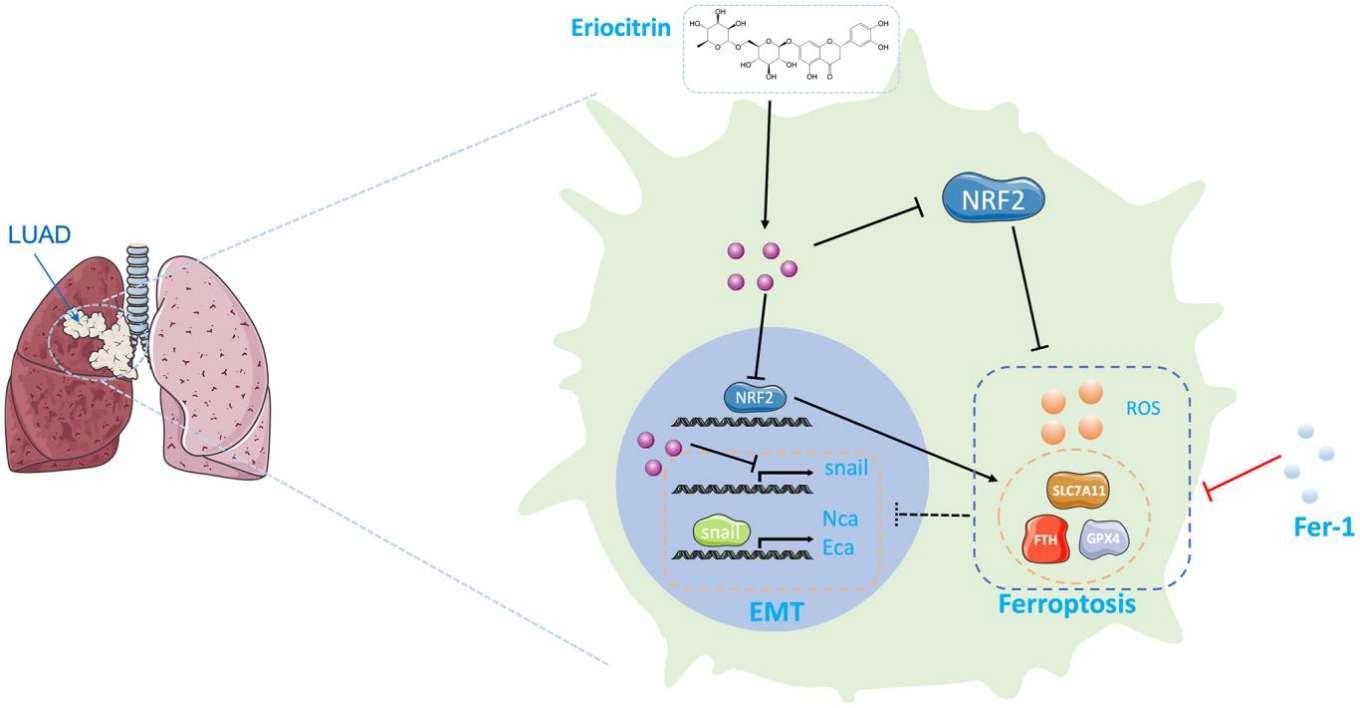
**Graphical abstract of the effects exerting by eriocitrin on lung cancer cells.** On the one hand, eriocitrin inhibits Snail expression to impede the EMT process of lung cancer cells. On the other hand, eriocitrin prevents Nrf2 expression to inhibit antioxidative genes expression, including GPX4, SLC7A11 and FTH1, thereby to increase cellular ROS to promote ferroptosis. Besides, inhibiting ferroptosis by Fer1 inverses the inhibitory effects of eriocitrin to EMT procedure. Abbreviations: EMT: epithelial-mesenchymal transition; Fer1: Ferrostatin1.

Overall, we proved for the first time that edible natural flavonoid, eriocitrin can effectively suppress the proliferation and EMT procedure of lung cancer cells *in vitro*. Also, we demonstrate that ferroptosis could be triggered by eriocitrin to depress EMT of lung cancer cells (Figure 6). Therefore, eriocitrin can be serve as a potential chemopreventive natural product for the treatment of LUAD.

## Supplementary Materials

Supplementary Table 1
