# Supplementary material for: Eriocitrin inhibits epithelial-mesenchymal transformation (EMT) in lung adenocarcinoma cells via triggering ferroptosis
Source: Aging (Albany NY). 2023 Oct 2;15(19):10089–104. doi: 10.18632/aging.205049 (PMC10599723; doi:10.18632/aging.205049)
Supplement: Supplementary Table 1 [file aging-15-205049-s001.pdf]

SUPPLEMENTARY TABLE

Supplementary Table 1. The primers sequence for used in this study.

Primer name	Primer sequence (5′–3′)
H-GAPDH-S	GGAAGCTTGTCATCAATGGAAATC
H-GAPDH-A	TGATGACCCTTTTGGCTCCC
H-CDH1-S	ATTGCTCACATTTCCCAACTCC
H-CDH1-A	CTCTGTCACCTTCAGCCATCCT
H-CDH2-S	GCCACCTACAAAGGCAGAAGAG
H-CDH2-A	CCTCAAATGAAACCGGGCTAT
H-Snail-S	TTTACCTTCCAGCAGCCCTA
H-Snail-A	GACAGAGTCCCAGATGAGCA
H-GPX4-S	GGCAAGACCGAAGTAAACTACAC
H-GPX4-A	ACATATCGAATTTGACGTTGTAGC
H-FTH1-A	ATTGCATTCAGCCCGTTCTC
H-FTH1-S	TTGGAAAGAAGTGTGAATCAGT
